# Pulmonary hypertension attenuates the dynamic preload indicators increase during experimental hypovolemia

**DOI:** 10.1186/s12871-017-0329-z

**Published:** 2017-03-03

**Authors:** Juan P. Bouchacourt, Juan Riva, Juan C. Grignola

**Affiliations:** 1 Department of Anesthesia, School of Medicine, Hospital de Clínicas, Universidad de la República, Montevideo, Uruguay; 2 Department of Pathophysiology, School of Medicine, Hospital de Clínicas, Universidad de la República, Avda Italia 2870, PC 11600 Montevideo, Uruguay

**Keywords:** Preload dynamic indices, Stroke volume variation, Pulse pressure variation, Pulmonary hypertension, Right ventricular dysfunction

## Abstract

**Background:**

Pulse pressure (PPV) and stroke volume (SVV) variations may not be reliable in the setting of pulmonary hypertension and/or right ventricular (RV) failure. We hypothesized that RV afterload increase attenuates SVV and PPV during hypovolemia in a rabbit model of pulmonary embolism (PE) secondary to RV dysfunction.

**Methods:**

Seven anesthetized and mechanically ventilated rabbits were studied during four experimental conditions: normovolemia, blood withdrawal, pulmonary embolism and fluid loading of a colloidal solution. Central venous, RV and left ventricular (LV) pressures, and infra-diaphragmatic aortic blood flow (AoF) and pressure were measured. SV was estimated by the integral of systolic AoF. We analyzed RV and LV function through stroke work output curves. PPV and SVV were obtained by the variation of beat-to-beat PP and SV, respectively. We assessed RV and LV diastolic and systolic function by the time rate of relaxation (tau) and the ratio of the first derivative of ventricular pressure and the highest isovolumic developed pressure (dP/dt/DP), respectively. The vasomotor tone was estimated by the dynamic arterial elastance (Ea_dyn_ = PPV/SVV).

**Results:**

PPV and SVV increased significantly during hemorrhage and returned to baseline values after PE which was associated to biventricular right-downward of the stroke work curves and a decrease of AoF and SV (*P* < 0.05). RV systo-diastolic function and LV systolic function were impaired. All the animals were nonresponders after volume expansion. Ea_dyn_ did not show any significant change during the different experimental conditions.

**Conclusions:**

The dynamic preload indicators (SVV and PPV) were significantly reduced after a normotensive PE in hypovolemic animals, mainly by the systo-diastolic dysfunction of the RV associated with LV systolic impairment, which makes the animals nonresponsive to volume loading. This normalization of dynamic preload indices may prevent the detrimental consequence of fluid loading.

## Background

Fluid therapy remains a highly debated topic in ICU patients as well as those undergoing high-risk surgery. The effective fluid management to prevent and treat hypo/hypervolemia is a very challenging task. Two groups of experts have recently highlighted the importance of fluid optimization guided by predefined therapeutic goals when patients require augmentation of their perfusion and are volume responsive [1, 2].

The concept that heart-lung interactions during intermittent positive-pressure ventilation could be used to predict fluid responsiveness was developed during the past decade [3]. Respiratory variation of stroke volume (SVV) or its surrogates, such as pulse pressure variation (PPV) have been demonstrated to predict preload responsiveness accurately in mechanically ventilated patients [4]. However, it soon became apparent that large confounders related to cardiac rhythm and function, respiratory mechanics, and ventilator settings can significantly limit the reliability of SVV and PPV in predicting fluid responsiveness. [5–8]. In an experimental model of acutely impaired cardiac function, Eichhorn et al. reported that functional parameters of cardiac preload allow prediction of fluid responsiveness [9]. However, recent studies suggest that PPV is not an accurate predictor of fluid responsiveness in subjects with pulmonary hypertension (PH) and/or right ventricular (RV) dysfunction [10, 11]. In the presence of RV failure, PPV and SVV are falsely elevated secondary to a RV stroke volume reduction during the inspiratory increase in RV afterload. In such cases, a high PPV or SVV could be a sign of RV afterload dependence rather than of fluid responsiveness. Therefore, the presence of RV failure should be suspected when a patient has large variations of SV and PP but does not respond to fluids [12, 13]. However, the effect of RV dysfunction on the dynamic preload indicators could be difficult to interpret depending on the study conditions (degree and type of PH, cause of the RV dysfunction), the study protocol (normo or hypovolemia before RV failure induction) and the definition of RV failure (RV ejection fraction, peak systolic velocity of tricuspid annular motion, systolic and/or diastolic dysfunction) [12, 14, 15].

We hypothesized that RV afterload increase might attenuate the increase of dynamic indices (SVV, PPV) during hypovolemia through the dysfunction of RV. We analyzed a) the effects of the RV afterload increase on preload dynamic indices and their ability to predict fluid responsiveness in a rabbit model of pulmonary embolism (PE) and b) the role of the right and left ventricular function by the stroke work output curves.

## Methods

This study conforms to the *Guide for the Care and Use of Laboratory Animals* published by the US National Institutes of Health (NIH Publication number 85–23, revised 1996). Ethical approval for this study (Ethical Committee number 070153-000363-13) was provided by the Ethical Committee Laboratory Animals of the Hospital de Clínicas, School of Medicine, Universidad de la República, Montevideo, Uruguay (CHEA -https://chea.edu.uy/﻿).

### Animal instrumentation

Seven female New Zealand rabbits (body weight 2.5 ± 0.1 kg) were included. Following intramuscular premedication with acepromazine (0.3 mg/kg) and meperidine (10 mg/kg), anesthesia was induced with a bolus dose of midazolam (0.5 mg/kg i.v.) and was maintained with a continuous infusion of midazolam (0.5–1 mg/kg/h) and rocuronium bromide (0.6 mg/kg/h) given via an ear vein. The animals were tracheotomized and mechanically ventilated (Amadeus Hamilton Medical AG, Switzerland) via an endotracheal tube (ID 2.5 mm) with a mixture of oxygen and room air. The ventilator was set in the volume controlled ventilation mode (tidal volume of 8 mL/kg, the end-expiratory pressure of 5 cmH_2_O, and a respiratory rate 38 ± 6 breaths/min). Heart rate/respiratory rate ratio was > 3.6. The end-tidal CO_2_ tension was monitored by capnography (Datex Inst Corp CD-200-43-00, Helsinki, Finland). Arterial blood gasses were measured at regular intervals (ABL520, Radiometer A/S, Brönshöj, Denmark) and the ventilation was adjusted to maintain normoxia and to avoid hypercapnia. The intravenous saline solution was administered at a rate of 7 mL/kg/h as maintenance requirements [6]. Normothermia was kept using a heating pad.

A 4.5F triple-lumen central venous catheter (Paediatric Multicath 3-Vygon) was placed into the left jugular vein for measuring central venous pressure (CVP), blood withdrawal, and pulmonary embolization. 20-G catheters were placed into the LV and RV through the right common carotid and right jugular vein to monitor LV and RV pressures, respectively. A non-constricting ultrasonic perivascular flow probe (2.5PSB-Series Flow probe, Transonic Systems Inc., Ithaca, NY, USA) was placed around the infra-diaphragmatic aorta by a right lumbar incision and an extra-peritoneum approach to measuring instantaneous aortic flow (Doppler flowmeter model T101, Transonic Systems Inc., Ithaca, NY, USA). Another 20-G catheter was advanced through the right femoral artery up to the infra-diaphragmatic aorta, just distal to the flow probe to monitor systemic arterial blood pressure. All pressure transducers (P23Db Gould Statham) were zeroed to atmospheric pressure and kept at the atrial level.

### Experimental protocol

After surgical instrumentation, the animals were allowed to stabilize for 30 min and all the variables described previously were recorded and stored during four conditions:

1. Normovolemia (BL).
2. Hypovolemia (BW): blood was progressively withdrawn with a total of 10 mL/kg of body weight (15% of volemia) by stepwise cumulative volumes of 5 mL/kg.
3. Pulmonary embolism (PE): thirty minutes after BW, we carried out PE with fresh autologous blood clots. One mL of blood clots were cut into 1- to 2- mm segments and suspended in 5 mL of saline solution. We progressively injected the suspended blood clots over 30 min until systolic RV pressure increase about 50% to compromise RV function secondary to the acute RV afterload increase [16].
4. Fluid loading (10 mL/kg) (VOL) of a colloidal solution (Voluven, Hydroxyethylstarch 6%, Fresenius Kabi, Germany) was finally produced.

Measurements of each experimental stage were obtained after a short period of stabilization. Once the experimental protocol was completed, the animals were euthanized with intravenous potassium chloride under deep anesthesia.

### Data acquisition and analysis

**Fig. 1:**
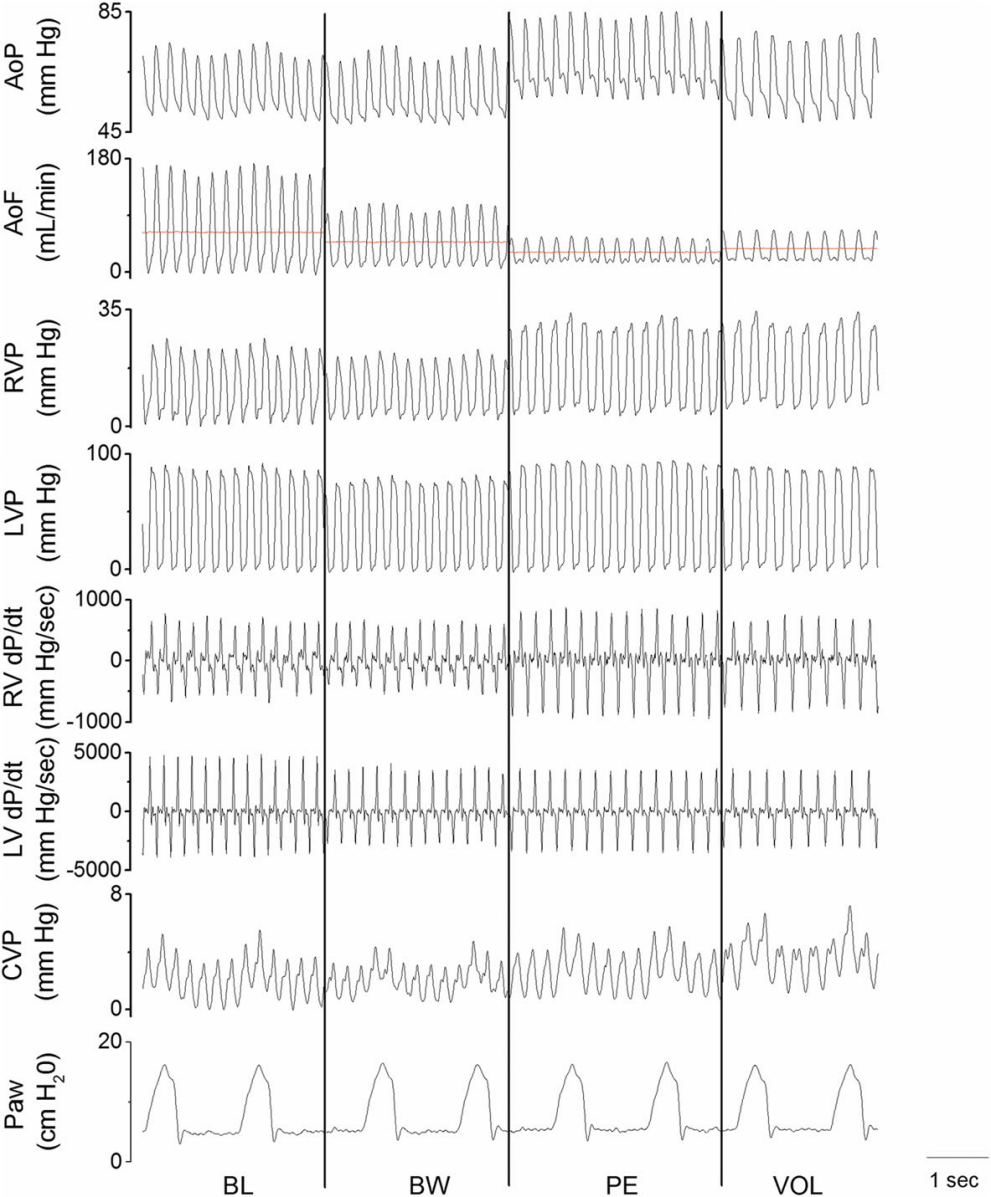
Raw data showing the different hemodynamic variables in a representative rabbit. AoF and AoP: aortic flow and pressure, respectively, CVP: central venous pressure, LVP and LV dP/dt: left ventricular pressure and its first derivative, respectively, Paw: airway pressure, RVP and RV dP/dt: right ventricular pressure and its first derivative, respectively. BL: baseline, BW: blood withdrawal, PE: pulmonary embolism, VOL: ﻿f﻿luid loading

All signals were monitored in real time (Fig. 1) and stored digitally with hardware and software specially designed in our laboratory (SAMAY M16). All measurements were taken at the end expiration with the ventilator turned off. Direct and derived invasive values were processed ‘off line’.

We calculated the integral of the systolic aortic flow curve to estimate the SV for each cardiac cycle [6]. We assessed the vasomotor tone by dynamic arterial elastance (Ea_dyn_ = PPV/SVV) [6, 17]. We also assessed the total systemic vascular resistance (TSVR = mean aortic pressure/mean aortic flow) and arterial capacitance (C = SV/arterial pulse pressure) [18]. We assessed the RV and LV preload by end-diastolic pressure (RVEDP, LVEDP) that allows the analysis of the RV and LV function through stroke work output curves as a function of left and right changes in filling pressure related to stroke work [19].

The first derivative of RV and LV pressure (dP/dt) was digitally obtained to estimate the ratio of ventricular dP/dt to the simultaneously highest developed isovolumic pressure (dP/dt/DP, ventricular contractility index) [20]. The developed ventricular pressure (DP) was calculated as the difference between instantaneous ventricular pressure and EDP during isovolumic contraction period [21].

We used the time constant relaxation (tau) as an index of the rate of ventricular relaxation (active diastolic function). Tau was calculated as the time constant of monoexponential pressure (P) decay during the period between the time point of dP/dtmin (Po) and the time point at which dP/dt reached 10% of the dP/dtmin value [22]. We normalized tau according to the period of cardiac cycle (T): Tau/T.

From the recorded aortic pressure and flow, the PPV and SVV were calculated offline. The calculus was performed over three consecutive respiratory cycles including five heartbeats each. Dynamic indices are defined by the relative difference in maximum and minimum values for pulse pressure (PP_max_/PP_min_) and SV (SV_max_/SV_min_) for PPV and SVV, respectively according to:

$$ \begin{array}{l}100\times \frac{\left({Q}_{max}-{Q}_{min}\right)}{\left[\left({Q}_{max}+{Q}_{min}\right)\right]/2}\\ {}\end{array} $$

where Q = PP and SV for PPV and SVV, respectively.

### Statistical analysis

The sample size was calculated based on preliminary experiments. Power analysis revealed a sample size of seven rabbits to detect a 40% effect in SVV and PPV (standardized difference of 1.5: target difference/standard deviation) for a level of significance of 0.05 and a power of 80% to be achieved [23]. Statistical comparisons were performed using statistics software (SPSS for Windows Version 18.0; SPSS Inc., Chicago, IL). Data are expressed as mean ± SD if normally distributed and as median (25th and 75th percentiles) for non-normally distributed data. Normally distributed data (Shapiro-Wilk test) were analyzed with a one-way analysis of variance for repeated measurements (ANOVA), and non-normally distributed variables were analyzed with Friedman repeated analysis of variance of ranks. When statistical significance level has been reached, parametric (Bonferroni test) and non-parametric (Wilcoxon signed ranks test) univariate comparisons were performed. A *P* value < 0.05 was considered statistically significant.

## Results

**Fig. 2:**
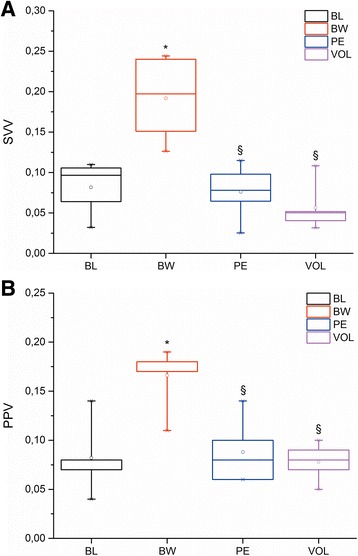
Box plots showing changes in A: stroke volume variation (SVV) and B: pulse pressure variation (PPV) during baseline (BL), blood withdrawal (BW), pulmonary embolism (PE) and volume loading (VOL). The line in each box indicates the median. The upper and lower limits of each box indicate the 75th and 25th percentiles, respectively. The error bars above and below each box represent the 90th and 10th percentiles, respectively. * *P* < 0.05 vs BL; § *P* < 0.05 vs BW

Tables 1 and 2 show the changes in baseline hemodynamic and right and left ventricular function data during BW, PE, and VOL, respectively. Hemorrhage (median blood volume loss, 24 ± 1.5 mL, ≈10 mL/kg) induced a significant increase of SVV and PPV (Fig. 2). On the contrary, RV and LV function (Table 2) and hemodynamic data (Table 1) did not show a significant change excepting the EDP decrease of both ventricles, reaching statistical significance only in LV (Table 2).

**Table 1:** Hemodynamic data during normovolemia (BL), hypovolemia (BW), pulmonary embolism (PE) and volume loading (VOL)

	BL	BW	PE	VOL
AoF (mL ^.^ min^-1^)	70 ± 8	53 ± 7	34 ± 10*	38 ± 10*
HR (bpm)	191 ± 37	180 ± 35	153 ± 37	152 ± 28
SV (mL)	0.26 ± 0.04	0.22 ± 0.05	0.14 ± 0.04*	0.16 ± 0.03*
AoP (mm Hg)	63 ± 5	62 ± 5	67 ± 6	62 ± 5
RVP (mm Hg)	20 ± 1	19 ± 2	30 ± 8*§	28 ± 5*§
TSVR (Wood unit)	0.9 ± 0.14	1.2 ± 0.2	2.1 ± 0.6*	1.7 ± 0.4*
C (mL ^.^ mm Hg^-1^ × 10^-3^)	9.3 ± 1.6	7.5 ± 1.7	4.5 ± 1.6*§	5.0 ± 0.8*
Ea_dyn_	1.1 (0.7, 1.3)	1.1 (0.8, 1.3)	1.2 (0.9, 1.8)	1.4 (0.9, 2.3)
CVP (mm Hg)	1.8 ± 0.7	0.13 ± 0.7	2.3 ± 0.9§	4.4 ± 1.6*§

Mean ± SD or median (25th, 75th percentiles). *p < 0.05 vs BL; §*p* < *0.05* vs BW. *AoF*, *AoP* aortic flow and pressure, respectively, *C* arterial capacitance, *CVP* central venous pressure, *Ea*_*dyn*_ dynamic elastance, *HR* heart rate, *RVP* right ventricular pressure, *SV* stroke volume, *TSVR* total systemic vascular resistance

**Table 2:** Right and left ventricular function data during normovolemia (BL), hypovolemia (BW), pulmonary embolism (PE) and volume loading (VOL)

	BL	BW	PE	VOL
Right ventricle				
EDP (mm Hg)	3.9 ± 0.8	2.2 ± 0.6	5.4 ± 2.1§	6.3 ± 1.9§
Tau (msec)	10 ± 7	9 ± 4	23 ± 5*§	27 ± 7*§
Tau/T (10^-3^)	2.1 ± 1.2	1.7 ± 0.8	4.9 ± 1.5§	6.0 ± 2.1*§
dP/dt/DP (sec^-1^)	91 ± 22	85 ± 21	65 ± 20*§	69 ± 17*
SW (mm Hg.mL)	3.2 ± 0.5	2.9 ± 0.7	2.4 ± 0.7	2.3 ± 0.8
Left ventricle				
EDP (mm Hg)	4.5 ± 1.5	1.3 ± 1.1*	4.0 ± 0.9	6.3 ± 1.8§
Tau (msec)	10 ± 6	9 ± 5	10 ± 4	11 ± 5
Tau/T (10^-3^)	2.6 ± 2.3	1.7 ± 1.0	2.1 ± 1.1	2.4 ± 1.5
dP/dt/DP (sec^-1^)	131 ± 13	126 ± 15	94 ± 16*§	99 ± 5*§
SW (mm Hg.mL)	15.0 ± 1.8	13.3 ± 3.0	9.0 ± 2.7*	9.5 ± 2.5*

Mean ± SD. *p < 0.05 vs BL; *§p* < 0.05 vs BW

*EDP* end-diastolic pressure, *dP/dt/DP* ratio of the first derivative of ventricular pressure to the simultaneously highest isovolumic developed pressure, *SW* stoke work; *tau* time constant relaxation, *Tau /T* ratio of tau and cardiac cycle period

**Fig. 3:**
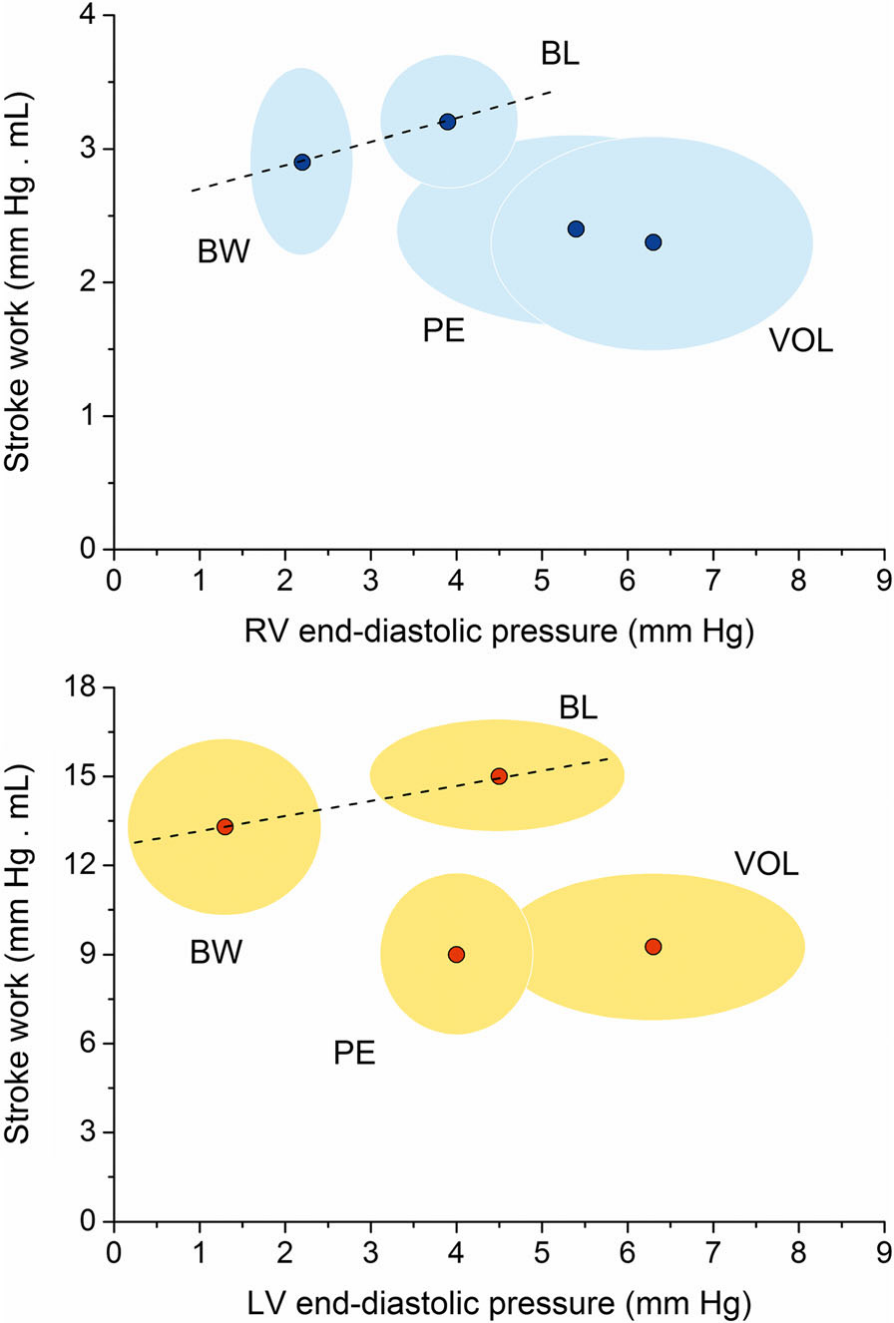
Right and left stroke work output function curves during baseline (BL), blood withdrawal (BW), pulmonary embolism (PE) and volume loading (VOL). Clouds have been drawn considering one standard deviation for each experimental condition

The total volume of the blood clot to reach 0.5 times the basal RV pressure was 0.25 mL ± 0.05 mL/kg. PE determined a significant decrease in end-tidal pressure of CO_2_ (35 ± 3 vs. 27 ± 2 mm Hg) which denotes an increase of alveolar dead space. The increase of RV afterload during PE determined that SVV and PPV significantly decreased with respect on BW, returning to baseline values in spite of the animals were hypovolemic (Fig. 2). Concomitantly, both ventricles operated on a flat right-downward Frank-Starling curve which reveals the impairment of the functional ability of the ventricles to pump blood (Fig. 3). RV active diastolic function (tau) and RV and LV systolic function (dP/dt/DP) were significantly impaired (Table 2), which was associated with a decrease aortic flow and SV (*P* < 0.05). RVEDP increased significantly in comparison with BW with a concomitant increase of CVP (*P* < 0.05). We discarded some possible effect of heart rate on the tau changes by the ratio tau/T.

All of the animals were nonresponders after volume expansion (VOL). RV aggravates its diastolic dysfunction showing a significant increase of CVP and RVEDP, with a non-significant further increase of the ventricular time constant relaxation maintaining an impaired systolic function (Table 2). Concomitantly, LVEDP increased significantly, with significant impairment of LV systolic performance (Table 2).

The dynamic arterial elastance did not show significant changes during the different experimental conditions, although TSVR increased, and C decreased during PE and after volume expansion (*P* < 0.05).

## Discussion

We demonstrated that the increase of RV afterload secondary to a normotensive PE abolishes the increment of the dynamic preload indices after bleeding. This was associated with RV global dysfunction and secondarily with LV systolic dysfunction, making the animals nonresponsive to volume administration. Although the normalization of PPV and SVV masks the true intravascular volume deficit, it would have prevented fluid loading which could either not improve SV and be detrimental.

We analyzed the position of the right and left ventricular function curves by plotting the ventricular stroke work against the filling pressure of each ventricle. Both ventricles showed a right downward change of the function curve position after PE and volume loading, operating on the flat part of the Frank-Starling curve and resulting in small cyclic variations of LV stroke volume and ultimately of SVV and PPV. Concomitantly, aortic flow and LV SV showed a significant decrease. Although the reduction of RV stroke work during PE did not reach statistical significance because of the concomitant increase of RV systolic pressure, the volume load did not reverse the ventricular performance, supporting the impairment of systolic function. The afterload increase induces a pronounced slowing of RV pressure fall that might lead to incomplete relaxation and therefore to an elevation of filling pressures [24]. The volume reposition maintained biventricular systolic impairment and aggravated RV diastolic dysfunction, impeding to improve aortic flow and LV SV, and, therefore, making LV nonresponsive to the volume challenge. The increase of RVEDP associated with a higher increase of CVP could explain the significant increase of LVEDP by a direct ventricular interaction [25]. It is well known that the function of the two ventricles is inextricably linked by both, series and parallel connections [26]. A moderate degree of acute PH secondary to PA constriction alters both the three-dimensional dynamic geometry and systolic function of the LV in conscious dogs [27, 28]. Acute RV dilation induced by right coronary artery occlusion produces a CO decrease due to a significant impairment of LV systolic performance related to the LV geometry distortion [29]. Accordingly, we observed a significant decrease in LV dP/dt/DP during PE, what could be interpreted as a compromise of the systolic function. Although LVEDP increases during VOL, the time constant of relaxation and tau/T did not significantly change. Probably, the concomitant LV underfilling secondary to PE (serial and parallel effects) could avoid a significant compromise of active LV diastolic function.

Previous studies have shown that SVV and PPV adequately predict fluid responsiveness [3, 4, 30]. Among the mechanisms responsible for the cyclic changes of LV SV during mechanical ventilation, the first two include the decrease in RV preload (due to an increase in pleural pressure) and the RV output impedance increase (secondary the increase in alveolar pressure) during inspiration that lead to a decrease in RV output and a subsequent decrease in LV output [31]. In the presence of RV failure and normovolemia, PPV and SVV may be falsely elevated mainly related to an inspiratory increase in RV afterload (and not to a decrease in RV preload) (false positive) [31]. Therefore, the presence of RV failure should be suspected when a patient has significant variations of SV or PP but does not respond to fluids [32]. Majhoub et al. hypothesized that the failing RV becomes more sensitive to afterload increase and is less affected by preload variation [12, 33]. During liver transplantation, Kim et al. reported that recipients with a RV ejection fraction ≤ 30% did not show significant increases in SVV or PPV despite having a cardiac output decrease ≥ 20% after inferior vena cava clamping [15]. Willer von Ballmoos et al. reported that patients with PH (cardiac surgery and septic shock) with the risk of acute RV dysfunction respond poorly to fluid administration. The fact that almost half of the nonresponders and none of the responders presented an impaired ejection fraction of RV would suggest that RV dysfunction is in part responsible for the poor predictive value of PPV [10]. In an acute PH experimental model (thromboxane-A2 infusion), Richter et al. showed that in the presence of RV dysfunction (defined as CVP increase, a decrease in RV ejection and CO), SVV and PPV remained high after volume removal, suggesting volume responsiveness. However, not all the animals increased CO after volume retransfusion. Therefore, the authors concluded that SVV and PPV failed to predict volume responsiveness. The responders animals had higher baseline CVP than nonresponders, which is difficult to explain since the more elevated CVP could be related to a worse RV function [14].

By contrast, in our experimental protocol, we first produced a mild hypovolemia to guarantee large respiratory variation in SV and PP previously to PE. Both, right and left ventricular dysfunction, “normalized” the dynamic indices after hemorrhage, making the animals nonresponsive to the volume administration (true negative). So, we could argue that in the setting of RV dysfunction, the performance of SVV and PPV could depend on the volemia status: during normovolemia their high values failed to predict volume responsiveness (false positive), by contrast during hypovolemia their normal values predict volume unresponsiveness (true negative), avoiding dangerous fluid loading. Consequently, in routine clinical practice, we should be aware of the limitation of SVV and PPV in fluid management whenever RV dysfunction is present.

Although TSVR increase and C decrease during PE and VOL, the unchanged of Ea_dyn_ allows to discard a significant change of vasomotor tone, and so, a possible role of the vasomotor tone on the normalization of SVV and PPV [6].

The present study has several limitations. Although the experimental protocol may not be common in clinical settings, it is not unusual to deal with a mild hypovolemic patient with some degree of RV dysfunction. The aim was to induce an increase of SVV/PPV by a mild hemorrhage (10 mL/kg) previously to PE to avoid false positive values.

We used the ratio dP/dt/DP to assess the ventricular contractility. However, it may be influenced to some extent by large changes in preload [20]. Peak values of dP/dt/DP were substantially independent of preload and afterload, except in the presence of extreme elevations of preload (EDP > 25 mm Hg) and afterload (aortic diastolic pressure > 120 mm Hg) that may decrease contractility [21].

Peripheral pulse pressure depends mainly on SV and arterial compliance. We only estimated the compliance of the abdominal aorta, not including ascending and descending aorta compliances. Nevertheless, the change of the abdominal aorta compliance could be representative of the compliance of the entire aorta since total compliance of a system is the sum of the individual compliances in series [34]. Flow probe only measures descending aortic flow (about 70%), excluding flow to the aortic arch vessels (30%). We assumed a constant diversion of blood flow during the different experimental conditions since the dynamic afterload (TSVR × C product) was maintained constant. Besides, the measure of the arterial pressure and flow at the same place could make the dynamic preload indexes assessed at abdominal aorta representative of the whole arterial system.

The gold standard of preload measurement is the end-diastolic volume. We used EDP which could be less representative since mainly depends on ventricular distensibility.

## Conclusions

The dynamic preload indicators (SVV and PPV) were significantly reduced after a normotensive PE during hemorrhage, primarily by the systo-diastolic dysfunction of the RV associated with LV systolic impairment, which makes the animals nonresponsive to volume loading. This normalization of dynamic preload indices may prevent the detrimental consequence of fluid loading.

## Abbreviations

BL: Baseline condition
BW: Blood withdrawal
C: Arterial capacitance
CO: Cardiac output
CVP: Central venous pressure
dP/dt/DP: Ratio of the first derivative of ventricular pressure to the simultaneous highest developed isovolumic pressure
Ea_dyn_: Dynamic arterial elastance
EDP: End-diastolic pressure
LV: Left ventricle
PE: Pulmonary embolism
PH: Pulmonary hypertension
PPV: Pulse pressure variation
RV: Right ventricle
SV: Stroke volume
SVV: Stroke volume variation
T: Cardiac cycle period
TSVR: Total systemic vascular resistance
VOL: Volume loading
Tau: Time constant relaxation
